# Supplementary material for: Structural Studies of Mexican Husk Tomato (Physalis ixocarpa) Fruit Cutin
Source: Molecules. 2023 Dec 28;29(1):184. doi: 10.3390/molecules29010184 (PMC10780591; doi:10.3390/molecules29010184)
Supplement: Supplementary file 1 [file molecules-29-00184-s001.zip › molecules-2752753-supplementary.pdf]

MONOMER I

9,10-EPOXY-18-HYDROXY-OCTADECANOIC ACID

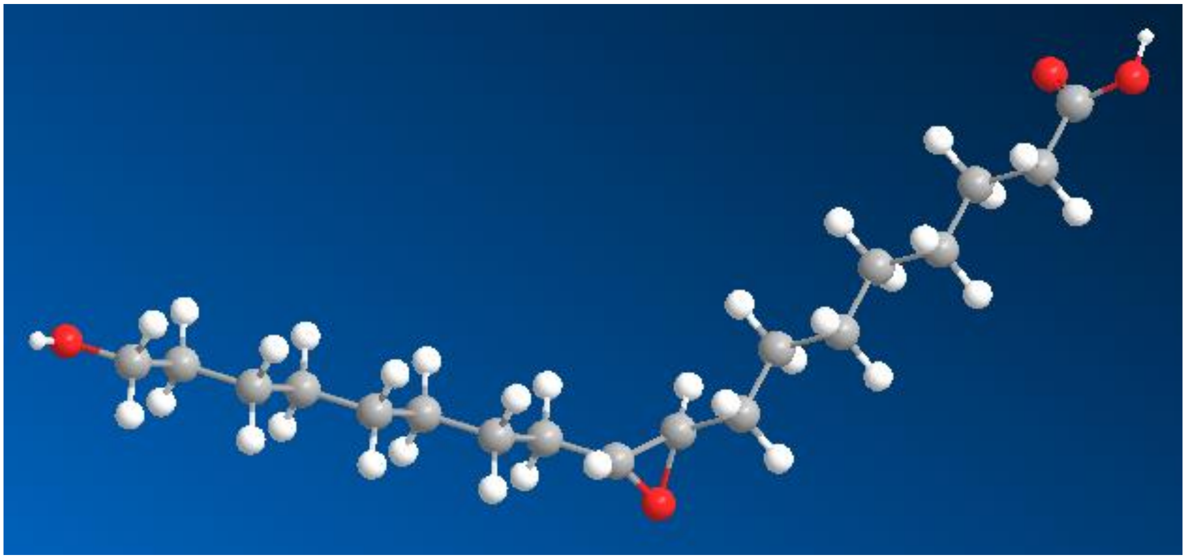

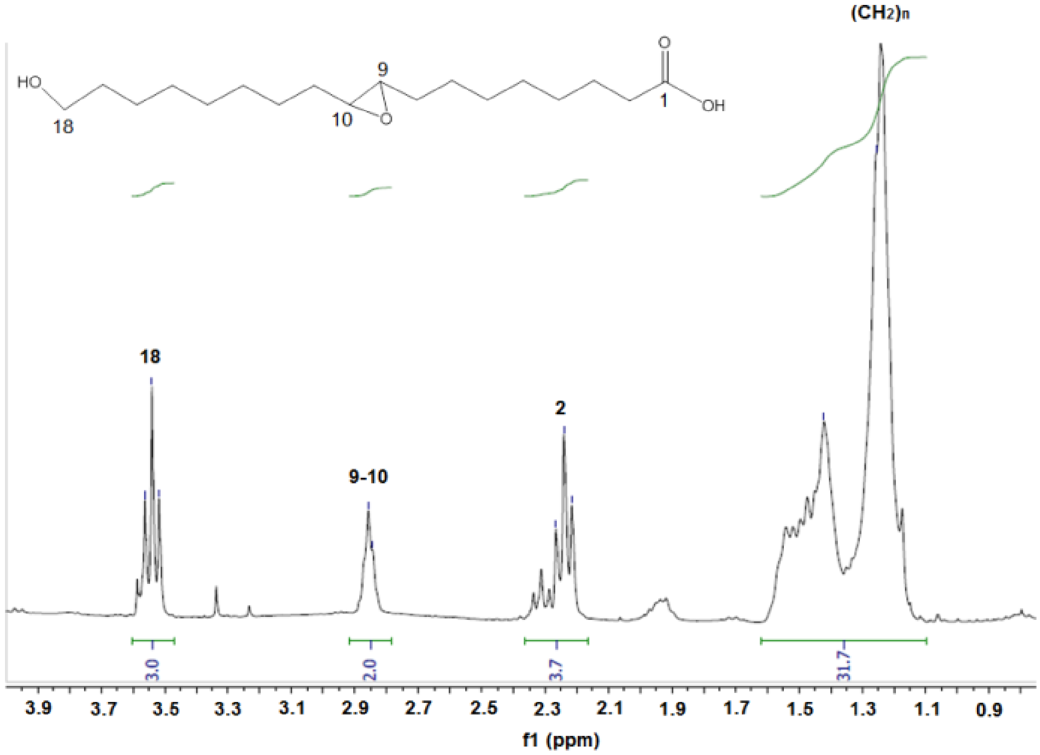

Figure S1. ^{1}H NMR spectra of 9,10-epoxy-18-hydroxy-octadecanoic acid.

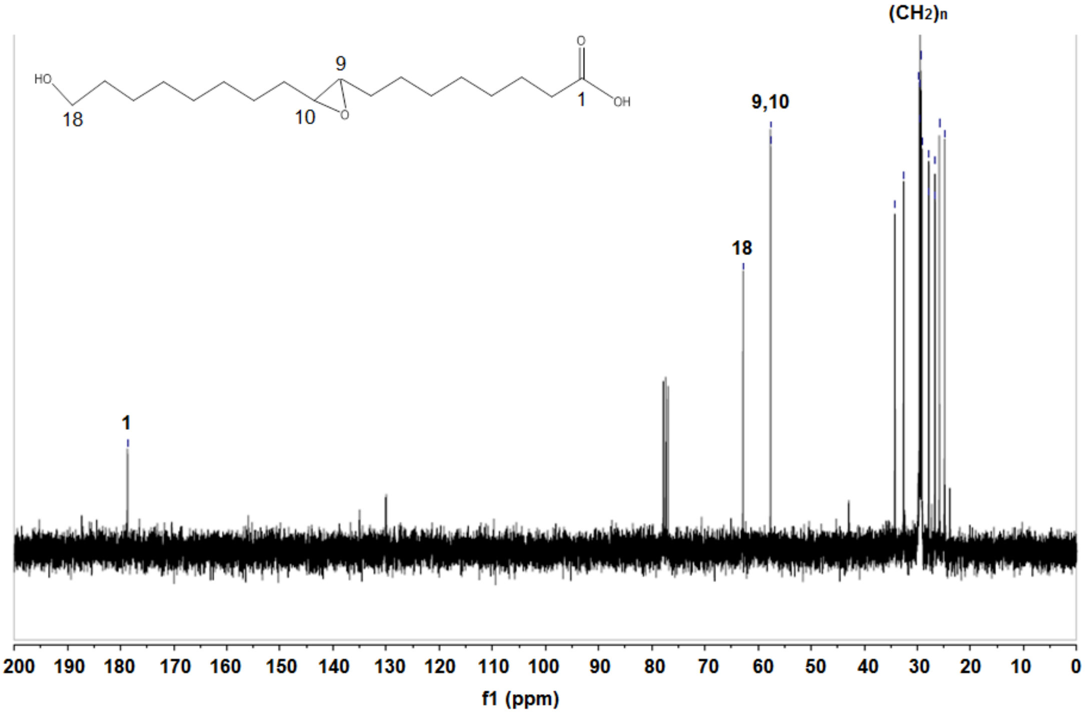

Figure S2. ^{13}C NMR spectra of 9,10-epoxy-hydroxy-octadecanoic acid.

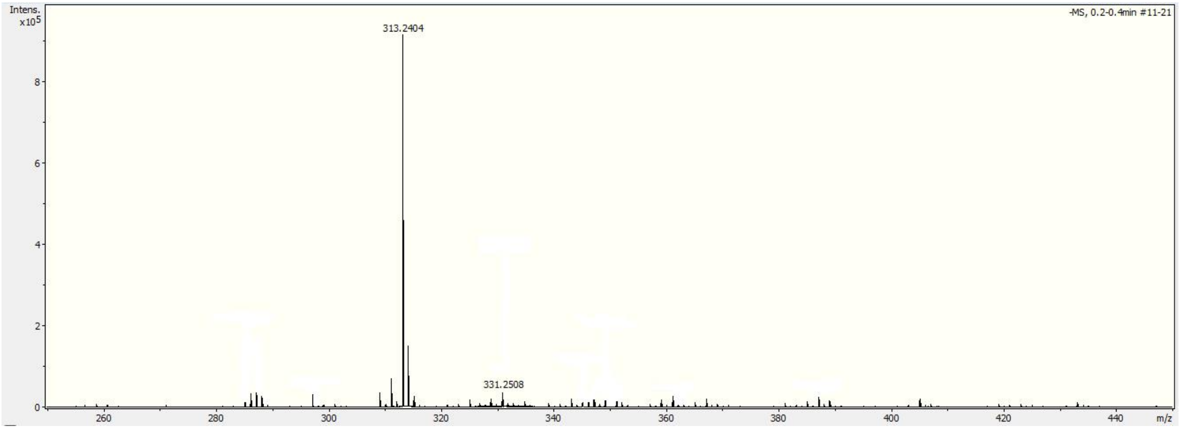

Figure S3. DIESI-MS spectra of 9,10-epoxy-hydroxy-octadecanoic acid.

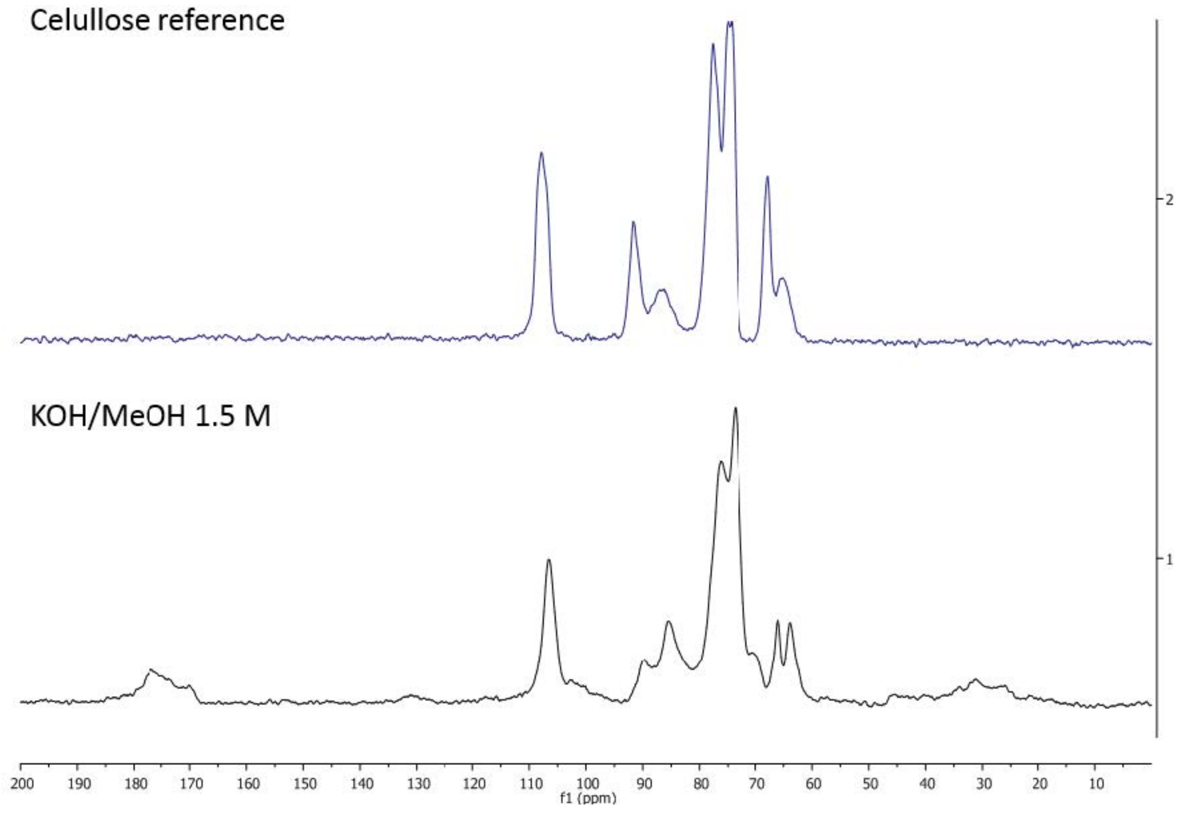

Figure S4. Comparative analysis of the cellulose (upper spectra) and residual cuticular material from the alkaline hydrolisis (KOH/MeOH 1.5 M)

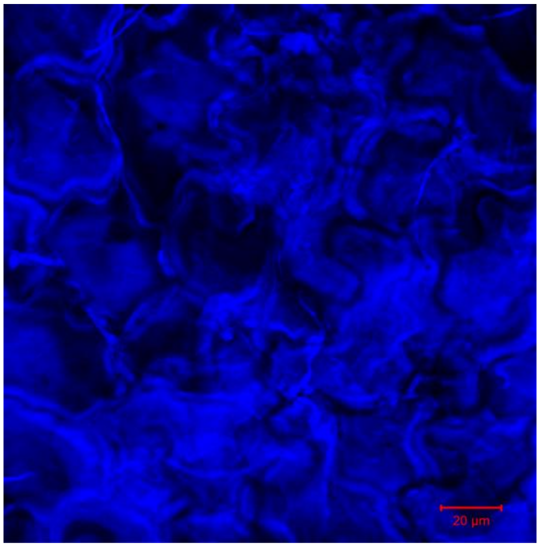

Figure S5. Cellulose fluorescence with calcofluor staining (405 nm) of the residual cuticular material from the alkaline hydrolisis (KOH/MeOH 1.5 M)
